# The use of clinical guidelines highlights ongoing educational gaps in physicians’ knowledge and decision making related to diabetes

**DOI:** 10.1186/1472-6920-14-186

**Published:** 2014-09-08

**Authors:** Mark D Corriere, Laura B Minang, Stephen D Sisson, Frederick L Brancati, Rita Rastogi Kalyani

**Affiliations:** Division of Endocrinology, Diabetes, & Metabolism, Johns Hopkins University School of Medicine, 1830 East Monument Street, Suite 333, Baltimore, Maryland 21287 USA; Division of General Internal Medicine, Johns Hopkins University School of Medicine, Baltimore, Maryland USA

**Keywords:** Diabetes, Clinical Guidelines, Education

## Abstract

**Background:**

Clinical guidelines for type 2 diabetes are a resource for providers to manage their patients and may help highlight specific areas in need of further education and training. We sought to determine how often guidelines are used and the relationship to physicians’ diabetes-related knowledge and decision making.

**Methods:**

Existing users of electronic clinical support tools were invited to complete an online questionnaire. A knowledge score was calculated for five questions related to prevention of diabetes and treatment of its complications. We explored the association of clinical guideline use with diabetes-related knowledge and self-reported decision making using logistic regression models, adjusted for key covariates.

**Results:**

Of 383 physicians completing the questionnaire, 53% reported using diabetes guidelines routinely. Mean diabetes knowledge score for guideline users (GU) was significantly higher than non-guideline users (NGU) (3.37 ± 0.072 vs. 2.76 ± 0.084; p < 0.001). GU were significantly more likely to report a good understanding of type 2 diabetes medications (OR = 2.99, 95% CI 1.95-4.61; p < 0.001). GU were less likely to report their unfamiliarity with insulin as an important barrier to early insulin use (OR = 0.41, 0.21-0.80; p = 0.007) and with pharmacologic options as a barrier to prescribing intensive multifactorial interventions (OR = 0.32, 0.17-0.58; p < 0.001). Associations remained significant after adjusting for physician specialty, practice volume and frequency diagnosing or treating diabetes patients.

**Conclusions:**

Significant gaps exist in diabetes-related knowledge and decision making among practicing physicians, as highlighted by clinical guideline use. The development of educational and training strategies to address these needs may ultimately improve outcomes for patients with diabetes and should be investigated in the future.

**Electronic supplementary material:**

The online version of this article (doi:10.1186/1472-6920-14-186) contains supplementary material, which is available to authorized users.

## Background

The number of persons with diabetes continues to rise, with about 29 million Americans or 9.3% of the U.S. population having diabetes [1]. Estimates project that this number will nearly double over the next 25 years [2, 3]. With the rising prevalence of diabetes, it is imperative that physicians be familiar with the currently recommended standards of care regarding the prevention, diagnosis and treatment of diabetes in order to not only appropriately manage patients with the disease but also reduce the public health impact of diabetes and its complications on society.

Consequently, there exists a need for physicians to be well-trained in the care of patients with diabetes. The best educational methods to achieve this competency are not clearly established. Internal medicine training programs may not fully achieve this important educational objective. A study by our group found that internal medicine residency programs across the U.S. only modestly improve diabetes-related knowledge during training and many residents continue to have significant knowledge gaps in the last year of their training program [4]. Another study sampled over 2,000 physicians about their training in diabetes management. Two-thirds of participants reported that their training did not adequately prepare them to optimize diabetes treatments and indicated a need for further training in all aspects of diabetes care [5]. Similar findings were reported in an earlier multicenter United Kingdom survey by the same investigators. In this study, relatively recent medical school graduates were surveyed. Less than a quarter of physicians reported having full confidence that they could diagnose diabetes and only 15% reported that they would always optimize glycemic control in their practice [6]. These studies suggest that current educational methods may result in significant gaps in physician knowledge of diabetes management at the completion of formal medical training.

Further, in clinical practice, many patients with diabetes are still not achieving suggested guideline goals [7, 8]. A recent study demonstrated suboptimal attainment of the American Diabetes Association’s recommended targets in the U.S. population [9]. Based on National Health and Nutrition Examination Survey data from 2007–2010, among persons with diabetes, only 52.5% achieved an HbA1c < 7%, 51.1% achieved a blood pressure <130/80 mmHg, 56.2% achieved an LDL <100 mg/dL, and only 18.8% achieved all three goals [10]. Thus, the recommended standards of care are currently not being attained for the majority of patients with diabetes and inconsistent use of clinical guidelines by health care providers may be one of the many potential reasons [11].

Clinical practice guidelines may serve as an educational resource for physicians to improve their knowledge of diabetes management and optimize patient outcomes in clinical practice. Many societies publish and regularly update clinical guidelines for diabetes, including the American Diabetes Association, European Association for the Study of Diabetes, and the American Association of Clinical Endocrinologists [7, 9, 12–14]. These guidelines synthesize available evidence-based information with expert opinion to develop consensus recommendations for health care providers. Although clinical guidelines for diabetes are intended as an important resource for providers to achieve the standard of care for their patients with diabetes, the relationship of guideline use to diabetes-related knowledge and decision making by providers has not been formally described in previous studies but can potentially help identify areas of need for further training and continuing diabetes education.

Thus, the objectives of our study were to: 1) identify the frequency of clinical diabetes guideline use in a cohort of practicing physicians; 2) explore the association of clinical guideline use with diabetes-related knowledge and decision making by providers; and 3) describe the degree to which these relationships are independent of providers’ specialty, practice size, and diabetes patient volume.

## Methods

### Study population

The Point-of-Care Information Technology (POC-IT) Guides are electronic evidence-based resources on various topics for practicing physicians to use in clinical decision making. As part of a generalized needs assessment prior to the development of a specialized electronic resource for diabetes, a survey was developed to identify areas of need for diabetes-related education among users in this existing database. The database was comprised of any health care provider that had previously registered for use of a POC-IT Guide resource. Respondents were queried about their frequency of use of consensus guidelines for diabetes management in their practice. Additionally, participants were asked to respond to a series of knowledge-based questions related to diabetes and its complications and questions related to their clinical decision making. There was no financial or other incentive for participants to complete the study questionnaire.

Questionnaire invitations were emailed to potential participants as part of a needs assessment in January 2011 and were completed by July 31, 2011. Invitations were sent to all registered users in the database (~80,000 users) without *a priori* knowledge of which of these registrants actually treated patients with diabetes in their practice and which registrants were physicians. In addition, we could not confirm that invitation emails were successfully received by users. Thus, to improve response rate, reminder emails were sent periodically to all users of the database. A total of 1,279 persons visited the survey website. Of these individuals, 655 participants fully completed the questionnaire and 238 participants partially completed the questionnaire and reported treating patients with diabetes in their practice. Of those who fully completed and submitted the questionnaire, 270 were other types of health care providers (nurses, podiatrists, dieticians, pharmacists, and other) and excluded. In the following study, we focused on the remaining physician respondents given that we were interested in exploring therapeutic decision-making by providers, as well. The database of registrants includes health care providers who manage multiple chronic diseases; diabetes is only one of these diseases. Also, other allied health professionals in addition to physicians were in the database. This partially accounts for what appeared to be a low overall response rate; nonetheless, the relatively large number of invitations was necessary to effectively recruit physicians that manage patients with diabetes for our study. Yet, the number of physician participants recruited for our study was still relatively large compared other studies exploring clinical guideline use [15, 16]. Among physician respondents that submitted the questionnaire, 2 participants had missing information for the question regarding the use of clinical guidelines, resulting in 383 participants for our study. All respondents care for patients with diabetes in their practice.

The study was approved by the Institutional Review Board at Johns Hopkins University School of Medicine.

### Questionnaire content

Respondents were asked in a multiple choice questionnaire about their familiarity with the most recent consensus guidelines for diabetes at the time including the American Association of Clinical Endocrinologists (AACE) roadmap and/or the American Diabetes Association/European Association for the Study of Diabetes (ADA/EASD) algorithm [7, 12]. Based on their selection of five possible answers, participants were dichotomized as guideline users or non-guideline users. Guideline users (GU) answered that “either the algorithm or the roadmap is deeply integrated into my practice” or “I have studied these guidelines and occasionally refer to them”. Non-guideline users (NGU) answered “I am unfamiliar with the specific content”, “I am aware they exist but they don’t apply to real world patient management”, or “I manage each patient as an individual and do not rely on these guidelines”. While the consensus guidelines referenced in the survey include sections for the management all types of diabetes, the focus of our study was on type 2 diabetes given that this represents the majority (>95%) of cases [1]. Thus, the survey questions focused primarily on the management of type 2 diabetes in adults although some of the diabetes-related knowledge questions may be common to other types. The Additional files 1 and 2 provide the full knowledge and clinical decision making questions that were included in this study which is in addition to the other questions described below regarding participant specialty, patient volume, and frequency treating patients with diabetes. Other survey questions related to management of prediabetes, suggested topics for future continuing medical education, or for which there were no clear consensus guidelines were not analyzed in our study. In total, the responses to 16 survey questions served as the basis for the results of our study.

Diabetes-related knowledge was assessed using five questions on key topics related to diabetes and its complications. These specific questions were chosen to identify areas of potential need for continuing diabetes education in the future. These questions assessed: [1] knowledge of the findings from the United Kingdom Prospective Diabetes Study, [2] recognition of the importance of early diagnosis and treatment in preventing complications, [3] identification of risk factors for diabetic foot ulcers, [4] knowledge of glycemic goals in critically ill patients, and [5] understanding the time course of progression from prediabetes to diabetes (Additional file 1). Each survey respondent received a “knowledge score” from 0 to 5 based on the number of these questions they answered correctly. Survey respondents that did not complete all five questions were excluded from the knowledge score assessment.

To characterize how physician participants’ use of clinical guidelines relates to management of patients with diabetes, the questionnaire also included multiple-choice questions about clinical decision making and use of diabetes therapies (Additional file 2). Physicians were asked about decisions related to use of early insulin and barriers to their implementation of multifactorial intensive treatments for patients. Lastly, physicians were asked about their familiarity with currently available diabetes treatments and use in their practice.

Physician participants also reported their specialty and characteristics of their clinical practice with multiple choice questions. Possible specialty responses were “primary care”, “endocrinology” or “other”. Practice volume was assessed with a multiple choice question about the number of patients seen on average each month in a physician’s outpatient practice. “High” volume practices were defined as physicians who reported > 250 patients each month (i.e. the participant selected one of the following options: 251–500, 501–750, or more than 750 patients) while “low” volume practices were defined as physicians who reported ≤ 250 patients each month (i.e. options selected were either less than 100 or 100–250 patients). The frequency of treating patients with diabetes was assessed with a number of questions. This included how frequently the physician made a new diagnosis of type 2 diabetes. A “high” frequency of making new diabetes diagnoses was defined as physicians who reported one or more new diagnoses a week (i.e. 1–3 times per week or more than 3 times per week). A “low” frequency of making new diabetes diagnoses was defined as physicians who reported less than one new diagnosis a week (i.e. less than once a week, less than once a month, or rarely if ever). Physicians were asked how often they treated patients with established diabetes in their practice. A “high” frequency of treating established diabetes patients was defined as physicians who reported ≥ 5 diabetes patients a week (i.e. 5–10 times per week, 11–20 times per week, or more than 20 times per week). A “low” frequency of treating established diabetes patients was defined as physicians who reported < 5 diabetes patients a week (i.e. less than 5 times per week or rarely if ever). Finally, physician participants were asked about how often they provided care to hospitalized patients with type 2 diabetes. The providers were dichotomized as “high” if they chose “often” or “very often” for treating hospitalized diabetes patients or “low” if they chose “never” or “seldom” for treating hospitalized diabetes patients.

### Statistical analysis

Clinical characteristics of guideline versus non-guideline users were compared using chi-squared test for binary outcomes. In addition, mean diabetes-related knowledge scores were compared using student’s *t* test for continuous outcomes. For purposes of analyses, and because there were fewer responses in the extreme categories, ordinal variables were categorized into dichotomous variables for the size of practice and frequency of diabetes care questions as described. Logistic regression models were created to explore the association of clinical guideline use with diabetes decision making both before and after adjustment for physician specialty, practice volume, and frequency diagnosing and treating patients with diabetes. All analyses were performed using Stata version 12 statistical software (College Station, Texas).

## Results

Based on responses to the questionnaire among 383 participants, 53% of the physicians were categorized as guideline users (GU) and 47% as non-guideline users (NGU). Baseline characteristics of GU vs. NGU are shown in Table 1. GU and NGU included a similar proportion of primary care physicians but there were more endocrinologists among GU (GU 7.7% vs. NGU 2.2%, p = 0.01) and more physicians from “other” subspecialties among NGU (NGU 51.1% vs. GU 36.1%, p = 0.003).

**Table 1:** **Clinical practice characteristics of guideline users vs. non-guideline users**

	Guideline users (n = 203)	Non-guideline users (n = 180)	p-value
**Specialty**			
Primary care	56.1%	46.7%	0.07
Endocrinology	7.7%	2.2%	0.01
Other	36.1%	51.1%	0.003
**Practice volume** ^*^			
High	35.6%	29.8%	0.23
Low	64.4%	70.2%	
**Frequency diagnosing diabetes** ^**^			
High	37.1%	22.8%	0.002
Low	62.9%	77.2%	
**Frequency treating patients with established diabetes** ^**†**^			
High	75.6%	67.0%	0.07
Low	24.4%	33.0%	
**Frequency treating hospitalized diabetes patients** ^**‡**^			
High	50.5%	51.1%	0.90
Low	49.5%	48.9%	

^*^High volume >250 patients a month, ^**^High frequency ≥ 1 diagnosis a week, ^†^High frequency ≥ 5 established diabetes patients a week, ^‡^High frequency treating hospitalized patients as “often” or “very often”.

Practice volume, frequency of treating patients with established diabetes and frequency of treating hospitalized patients with diabetes was similar among both groups. A larger proportion of GU reported diagnosing diabetes at a high frequency (37.1% of GU vs. 22.8% of NGU, p = 0.002).

Overall, the mean diabetes knowledge score was higher among GU versus NGU (3.37 vs. 2.76, p < 0.001, n = 364). Among individual questions, only the question about risk factors for diabetic foot ulcers had a similar proportion of correct responses among GU versus NGU. Otherwise, GU had a significantly higher proportion of correct responses to all of the knowledge questions compared to NGU. Specifically, three-quarters of GU correctly identified that the UKPDS demonstrated a decrease in microvascular complications while only half of NGU correctly answered this question (p < 0.001). Further, 34.3% of GU compared to 24.1% of NGU correctly identified a target glucose goal range of 140–180 mg/dl among critically ill patients (p = 0.03). Also, GU were more likely than NGU to answer correctly that early diagnosis and treatment of diabetes can help prevent complications (GU 78.4% vs. 66.8%, p = 0.01) and that prediabetes is commonly present for a few years (>24 months) before it progresses to diabetes (GU 54.6% vs. NGU 44.4%, p = 0.046).

Overall, 67.3% of GU reported that they “somewhat” or “completely” understood the available diabetes medicines in their practice while only 40.7% of NGU reported a similar understanding of diabetes medications in their practice (p < 0.001).

A similar proportion of GU versus NGU reported frequently prescribing early insulin treatment (i.e., more than 20% of the time) in patients on 1–2 oral agents with a hemoglobin A1c (HbA1c) >8% (GU 36.5% vs. NGU 28.2%, p = 0.09; n = 367). When asked about barriers to early initiation of insulin therapy in their practice, both groups had similar responses regarding patient factors (i.e., patient resistance, patient concerns about dosing regimens, inadequate resources for patient education and monitoring, and patients’ perception that insulin therapy represents disease progression). However, about a quarter of NGU reported that their own unfamiliarity with insulin was a significant barrier to early initiation (selected within the top three barriers) while a significantly smaller proportion of the GU group identified this as a significant barrier to early initiation of insulin therapy (GU 12.7% vs. NGU 26.1%, p = 0.009).

Participants were also asked to report reasons they might not adopt an intensive, multi-factorial approach to diabetes care, such as intensive lipid, blood pressure and glycemic control. A larger proportion of NGU reported unfamiliarity with pharmacologic options as one of the reasons they would not adopt an intensive multifactorial approach in their practice (GU 8.3% vs. NGU 22.2%, p < 0.001).

Table 2 displays logistic regression models exploring the relationship of clinical guideline use to diabetes-related knowledge and clinical decision-making. In unadjusted models (model 1), GU versus NGU were three times more likely to report a good (“completely” or “somewhat”) understanding of currently available diabetes medications than NGU (OR = 2.99, 95% CI 1.95-4.39, p < 0.001; n = 362); participants who chose “none of the above apply to my practice” were not included in this analysis. Among those participants that ranked all potential barriers to early insulin initiation, GU compared to NGU were 59% less likely to indicate unfamiliarity with insulin as an important barrier to early insulin initiation (OR = 0.41, 0.21-0.80, p = 0.007, n = 243) and 68% less likely to report unfamiliarity/inexperience with pharmacologic options as a reason for not adopting an intensive multifactorial approach (blood pressure treatment, lipid treatment, glucose control) (OR = 0.32, 0.17-0.58, p < 0.001, n = 371). All these findings were largely unchanged and remained statistically significant in adjusted logistic regression models that accounted for physician specialty, practice volume and frequency diagnosing and treating patients with diabetes (model 2).

**Table 2:** **Logistic regression models (Odds ratios and 95% confidence intervals) exploring the relationship of clinical guideline use to diabetes-related knowledge and clinical decision making**

Physician responses	Unadjusted model	Adjusted model ^*^
Good understanding of diabetes medications in their practice	2.99 (1.95-4.61)	2.80 (1.79-4.39)
Unfamiliarity with insulin as a barrier to early insulin initiation	0.41 (0.21- 0.80)	0.37 (0.18-0.76)
Unfamiliarity/inexperience with pharmacologic options as a barrier to adopting intensive multi-factorial interventions	0.32 (0.17- 0.58)	0.33 (0.17-0.62)

*Adjusted for physician specialty, practice volume and frequency diagnosing and treating patients with diabetes.

In sensitivity analysis including only primary care physician respondents (n = 199), we observed similar associations regarding knowledge scores and clinical decision making. Specifically, diabetes related knowledge scores remained significantly higher among GU versus NGU. Physician unfamiliarity/inexperience with pharmacologic options was also more commonly reported as a barrier to adopting intensive multi-factorial interventions among NGU compared to GU, and the differences remained statistically significant.

## Discussion

In the present study, we report that significant gaps in diabetes-related knowledge and familiarity with diabetes therapies exist in practicing physicians. Strikingly, there existed a low level of diabetes-related knowledge among both guideline and non-guideline users. However, clinical guideline use was associated with significantly better diabetes-related knowledge. Also, guideline users reported greater familiarity with currently available treatments for diabetes and clinical decision making that was more consistent with currently recommended standards of care, independent of specialty and clinical practice characteristics. The demonstrated differences in diabetes-related knowledge and clinical decision making according to use of clinical guidelines helps highlight important areas of ongoing educational need among practicing physicians.

Clinical guidelines may serve as an educational resource for health care providers who manage patients with diabetes. The use of clinical guidelines as educational tools has been investigated for general medical conditions. Grimshaw and colleagues performed a systematic review to assess the impact of clinical guideline use on general medical practice [17]. He concluded that explicit guidelines do improve clinical practice but with considerable variations in performance. Other educational tools that improve knowledge have also been shown to positively affect clinical outcomes in small studies of physicians who care for patients with diabetes [18, 19]. The DIABEDS trial demonstrated that a physician education program among internal medicine residents could lead to objective improvements in fasting glucose, HbA1c, and body weight among their patients [20]. Similar findings have been shown among practicing community physicians who, in another study, participated in a physician education project and subsequently demonstrated an improvement in the frequency of HbA1c testing and, eventually, overall mean HbA1c values among their diabetes patients [21]. Our study only focused on surrogates of diabetes knowledge and decision making patterns. We did not attempt to demonstrate improved patient outcomes from use of clinical guidelines. Further interventional studies are needed to explore if clinical guidelines and/or other possible educational tools (i.e., diabetes curriculum development for medical trainees or continuing medical education programs for practicing physicians) can address the knowledge gaps identified by our study and ultimately improve outcomes for patients with diabetes.

There may be many potential pathways by which clinical guideline use, in particular, could be associated with improved diabetes-related knowledge and decision making. It is possible that the guidelines themselves impart physician readers with improved knowledge and decision making skills. In fact, we found that clinical guideline users had higher diabetes-related knowledge scores compared to non-guideline users in our study. Physician specialty and practice characteristics may also be contributing factors. Specialists (i.e., endocrinologists) may be more likely to adhere to guidelines published by their professional societies than primary care physicians [11]. Physicians that see more patients with diabetes may become more adept at caring for such patients and/or have a stronger need to be up-to-date with clinical guidelines for this disease. However, we found that associations of clinical guideline use with better diabetes-related knowledge and decision making persisted even after accounting for physician specialty and clinical practice characteristics.

The strengths of our study include the relatively large number of participants examined and inclusion of both primary care and specialist physicians, in comparison to other studies on clinical guideline use [15, 16]. We also assessed a spectrum of disease-specific knowledge from diabetes prevention and treatment to management of long-term complications. In addition, we investigated the relationship of clinical guideline use to both gaps in physician knowledge and also clinical decision making which further extended our findings and have not previously been reported in the literature. Finally, we were able to examine providers’ familiarity with currently available diabetes treatments such as early initiation of insulin therapy and implementation of multifactorial interventions that remain relevant to modern practice and guidelines [9, 14].

Our study has limitations. First, the cross-sectional design does not allow us to infer temporality. Indeed, the reverse association is possible; physicians with improved baseline knowledge may seek out educational resources and be more likely to use clinical guidelines. However, we were interested in the association of clinical guideline use with diabetes-related knowledge and decision making, as clinical guidelines may represent an educational tool that could be used in the future. We dichotomized participants as guideline users versus non-guideline users based on their responses; only those that reported using clinical guidelines in their practice were included in the guideline user category. It is possible that some unrecognized familiarity of guidelines was also present in the NGUs but this would have overestimated knowledge scores of this group and limited our ability to detect associations. Also, residual confounding is possible. We did not have information about physicians’ demographic characteristics or geographic location of practice. However, previous studies have had mixed results and not consistently demonstrated that these factors are related to clinical guideline use in other specialties [22, 23]. Characterizing the individual participant characteristics associated with diabetes clinical guideline use was not the focus of our study, but may be interesting to examine in the future. In addition, physicians’ attitudes or motivation due to other factors may influence decision making, irrespective of their use of clinical guidelines [24, 25], yet, we still found significant relationships in our study. The inclusion of endocrinologists as well as primary care physicians may have resulted in a more heterogeneous study population and limited power; however, we still detected significant associations and found that results were similar when restricted to only primary care physicians in sensitivity analyses suggesting consistency of associations within each specialty. Also, the knowledge score was not designed to be a comprehensive assessment of a participants’ diabetes-related knowledge but instead to identify areas of potential educational need. The source of our study sample may also represent a limitation. All subscribers to an online clinical decision-making program were invited to participate without *a priori* knowledge of which registrants of the database routinely manage patients with diabetes. There was no financial or other incentive for participants to complete the study questionnaire. The database of registrants includes other providers in addition to physicians who manage multiple chronic diseases; diabetes is only one of these diseases. This partially accounts for what appeared to be a low overall response rate; yet, the relatively large number of invitations was imperative in order to recruit an adequate number of physicians that care for patients with diabetes which was comparatively larger than other studies [15, 16]. Consequently, selection bias may limit generalizability of our findings.

The survey tool we utilized has not yet been validated but should be more critically examined in studies specifically designed for this purpose. However, our study nonetheless provides exploratory findings that can be further investigated in representative populations of diabetes providers in the future. Given the absence of other literature regarding current gaps in diabetes-related knowledge among practicing physicians or the relationship of diabetes clinical guideline use to providers’ familiarity with diabetes therapies and clinical decision making, our study remains informative and provides findings that have not been previously reported.

## Conclusion

Our study demonstrates a significant gap in diabetes-related knowledge and familiarity with existing therapies among practicing physicians. The use of clinical diabetes guidelines as a potential educational resource was associated with better diabetes-related knowledge and clinical decision making that was more consistent with consensus recommendations among physicians in our study. The clinical care of patients with diabetes often uses a team care approach involving not only the physician but also nurse practitioners, physician assistants, practice facilitators, and medical assistants who may also refer to consensus guidelines. Future studies should explore potential knowledge gaps in these other specialties. Nonetheless, the findings of our study suggest the need for improved continuing medical education among practicing physicians and potentially training in diabetes management and treatment. Intervention studies that investigate the use of clinical guidelines and other possible educational tools, both during and following medical training, on diabetes-related knowledge, clinical decision making, and, ultimately, patient outcomes can help inform education-based strategies for improving diabetes care in the future.

## Electronic supplementary material

Additional file 1: **Diabetes-Related Knowledge Assessment Questions.** (DOC 28 KB)

Additional file 2: **Questionnaire of Physicians’ Clinical Decision Making and Management of Diabetes.** (DOC 30 KB)
