# Supplementary material for: The use of clinical guidelines highlights ongoing educational gaps in physicians’ knowledge and decision making related to diabetes
Source: BMC Med Educ. 2014 Sep 8;14:186. doi: 10.1186/1472-6920-14-186 (PMC4162949; doi:10.1186/1472-6920-14-186)
Supplement: Supplementary file 1 — Additional file 1: Diabetes-Related Knowledge Assessment Questions. (DOC 28 KB) [file 12909_2014_1009_MOESM1_ESM.doc]

**Additional file 1: Diabetes-Related Knowledge Questions**

1. How important is early diagnosis and prompt initiation of treatment with regards to long-term outcomes in Type 2 Diabetes?
2. There is no evidence to formulate and opinion
3. I am unaware of any such association
4. I suspect it doesn’t make much difference over the long haul
5. Early diagnosis and treatment may favorably affect some patients
6. Early diagnosis and treatment can prevent serious diabetic complications

*Correct answer e.*

1. The United Kingdom Prospective Diabetes Study showed patients who received intensive blood glucose control had:
2. No change in microvascular complications
3. A decrease in microvascular complications
4. An increase in microvascular complications
5. An increase in macrovascular complications
6. I am not familiar with this study

*Correct answer b.*

1. Important risk factors for developing diabetic foot ulcers include:
2. Peripheral arterial disease
3. Diabetic Neuropathy
4. Prior foot ulcers
5. All of the above
6. None of the above

*Correct answer d.*

1. For the majority of critically ill patients, the ADA and AACE recommend a blood glucose target of:
2. Less than 100 mg/dL
3. 100 to 140 mg/dL
4. 140 to 180 mg/dL
5. 180 to 200 mg/dL
6. Anything less than 200 mg/dL

*Correct answer c.*

1. On average, most patients with Type 2 Diabetes have had prediabetes for how long prior to clinical presentation?
2. 30 days or less
3. 90 days or less
4. 12 months or less
5. 12-24 months
6. Greater than 24 months

*Correct answer e.*
