# Supplementary material for: The use of clinical guidelines highlights ongoing educational gaps in physicians’ knowledge and decision making related to diabetes
Source: BMC Med Educ. 2014 Sep 8;14:186. doi: 10.1186/1472-6920-14-186 (PMC4162949; doi:10.1186/1472-6920-14-186)
Supplement: Supplementary file 2 — Additional file 2: Questionnaire of Physicians’ Clinical Decision Making and Management of Diabetes. (DOC 30 KB) [file 12909_2014_1009_MOESM2_ESM.doc]

**Additional file 2: Questionnaire of Physicians’ Clinical Decision Making and**

**Management of Diabetes**

1. For what percentage of your patients on 1-2 oral agents and uncontrolled Type 2 Diabetes (i.e. A1c>8%) have you prescribed early insulin therapy?
2. None
3. Less than 5%
4. Between 5% and 10%
5. Between 10% and 20%
6. Over 20%
7. In your practice, please rank the following barriers to early initiation of insulin therapy in management of Type 2 Diabetes:
8. Patient resistance (family experiences, aversion to needles)
9. My unfamiliarity with insulin use for Type 2 Diabetes
10. Patient concern over expected weight gain
11. My concern over expected weight gain
12. Patient concern over dose preparation and pre-meal injection
13. My concern over dose preparation and pre-meal injection
14. My satisfaction with oral antidiabetic drugs
15. Inadequate resources for patient education/monitoring
16. Affordability of insulin and supplies
17. Insulin signals disease progression to patient
18. In your practice, which of the following factors dissuade you from adopting an intensive multifactorial intervention approach (i.e. glucose, lipid and blood pressure control) for all your Type 2 Diabetes patients?
19. Risk of hypoglycemic events
20. Patient finances
21. Poor patient compliance
22. Inadequate patient education resources
23. Increased number of patient visits
24. Increased duration of patient visits
25. Unfamiliarity/inexperience with pharmacotherapeutic options
26. Not convinced of validity of published clinical trial findings
27. Most of my patients are satisfied with current approach
28. None of the above apply
29. In your practice, how would you describe your current understanding (i.e. indications, mechanisms, side effects) of available pharmacotherapeutic options for patients with Type 2 Diabetes?
30. I completely understand all Type 2 Diabetes treatment categories
31. I somewhat understand all Type 2 Diabetes treatment categories
32. I rely on a short list of Type 2 Diabetes medications that usually work for my patients and disregard the rest
33. I need to review the latest advances in antidiabetic pharmacotherapy
34. None of the above apply to my practice
